# Study of a Broadband Difference Interferometer Based on Low-Cost Polymer Slab Waveguides

**DOI:** 10.3390/nano9050729

**Published:** 2019-05-11

**Authors:** Kazimierz Gut

**Affiliations:** Department of Optoelectronics, Faculty of Electrical Engineering, Silesian University of Technology, 2 Krzywoustego Str., 44-100 Gliwice, Poland; kazimierz.gut@polsl.pl; Tel.: +48-32-2372182

**Keywords:** interferometer, single-mode optical waveguide, sensor, polymer waveguide

## Abstract

A model and the waveguide parameters of a broadband, polymer-based slab waveguide difference interferometer is presented in this paper. The parameters were determined based on knowledge of the dispersion in the structure materials used to fabricate the waveguide. The impact of the waveguide layer thickness, propagation path length, and change in the waveguide cover refractive index on the output signal from the system was determined. It has been shown that the direction of the maximum shifting is determined by the thickness of the waveguide layer. A relationship describing the shift in the signal extrema for a change in the waveguide cover refractive index was derived. The results show that the use of a propagation constant simplifies the description of the interferometer. Polymer waveguides, although they have a small contrast in refractive indices, allow for large shifts in the maxima of the signal. The determined shifts in the output signal extrema for polymer waveguides are comparable, and these shifts are larger for some waveguide thicknesses compared to waveguides based on Si_3_N_4_.

## 1. Introduction

Optical, label-free detection methods for applications in the biochemical field have been intensively investigated in recent years [1,2]. Systems of this type have remarkably advantageous properties, such as outstanding sensitivity, detection limit, wide dynamic range, and immunity to interference [3]. There are various methods for detecting effective refractive index changes such as surface plasmon resonance (SPR), microring resonators, reflectometric interference spectroscopy (RifS), and planar waveguide interferometers [4,5]. Mach–Zehnder and Young planar interferometers, which use a single wavelength, are the most sensitive systems in the latter group [1,4]. They can be used to detect changes to the effective refractive index in the order of 10^−8^.

A division of waveguide interferometers into common and double-path types was proposed in [4,6]. Mach–Zehnder and Young interferometers are typical double-path interferometers. In these interferometers, light propagates along two separate paths (sensor and measurement). Common-path interferometers are systems which have different types of modes (TE, TM), or modes of a different order with different sensitivities to changes in the system parameters, and which propagate along the same path. The impact of a change in the waveguide cover refractive index on the phase velocities of the propagated modes is most often used in these types of structures. The phase difference between modes at the output of the structure and the resulting intensity of light are a function of the waveguide cover refractive index. The mechanism presented is the basis for constructing common-path interferometers [7]. Interference between TE_0_ and TM_0_ modes is described in the literature [8,9,10,11,12]. Interference between the modes with different orders was also reported by [13,14]. The impact of changes in the cover refractive index on the mode field distribution in a two-mode structure is described in [15]. Common-path (single-channel) waveguide interferometers are often called difference interferometers.

Broadband interferometers were developed recently. Light from a selected spectral range propagates in these structures. In the case of monochromatic interferometers, phase ambiguity is a problem that is often encountered, which can be eliminated by using a broadband light source [5]. A description of the broadband Mach–Zehnder interferometer can be found in the literature [16,17]. The specific applications of this interferometric system can be found in many publications [18,19,20,21,22,23,24,25,26].

The broadband Young interferometer enables simultaneous measurement of the phase signal for two polarizations over a broad range of wavelengths [27]. The implementation of this interferometric system was discussed in a prior publication [28].

An analysis of the broadband difference interferometer was presented in [29]. In the aforementioned broadband interferometers, Si_3_N_4_ was used as a waveguide layer and SiO_2_ was used as a substrate. These types of structures have high contrast in the refractive index, resulting in a high sensitivity to changes in the system parameters. This paper presents a model describing a broadband difference interferometer based on the SU-8 polymer. In [30], a broadband planar SU-8 polymer-based difference interferometer was analyzed for a waveguide layer with a single selected thickness. In this case, interference maxima shift toward short waves (so-called blue shift). The results in this paper demonstrate the significant impact of the polymer waveguide layer thickness on the output signal from the interferometer. Depending on the thickness of the waveguide layer, it is possible to obtain the shift of the interference maxima towards short or long waves (so-called blue or red shift). The value of the maximum shift is also dependent on the thickness of the waveguide layer. In this paper, it is shown that the increase in the propagation path in the structure results in the increase in the number of maxima in the output signal. The characteristics presented in the paper allow optimization of the waveguide structure for practical applications. This type of research has not been conducted for polymer waveguides with a relatively low contrast in the refractive index in the visible light range.

## 2. Materials and Interferometer System

Polymer waveguides provide a low-cost alternative to Si_3_N_4_-based waveguides. This type of waveguide, is used for transmission between electronic circuits due to the possibility of obtaining relatively low attenuation [31]. Many interferometric structures are designed and implemented using various types of polymers. SU-8 is a common polymer used in this application [32,33].

Initially, this material was developed to meet the needs of the semiconductor industry, which required progressively more sensitive and cheaper photosensitive substances [34]. It was quickly found that the unique properties of SU-8 could also be used to fabricate MEMS and MOEMS. Interest in this polymer in terms of its use in sensor technology has risen considerably [35]. The possibility of thermal modification of the refractive index of the SU-8 layers was shown in [36]. Nowadays, research aims at reducing the hydrophobicity of the polymer surface [37]. There is a possibility of direct laser writing on the SU-8 layer, yielding low-loss waveguide structures [38]. Free-standing waveguides with higher refractive index contrast were also presented [39]. Sensors based on these structures have a high sensitivity to changes in the refractive index. 

**Figure 1:**
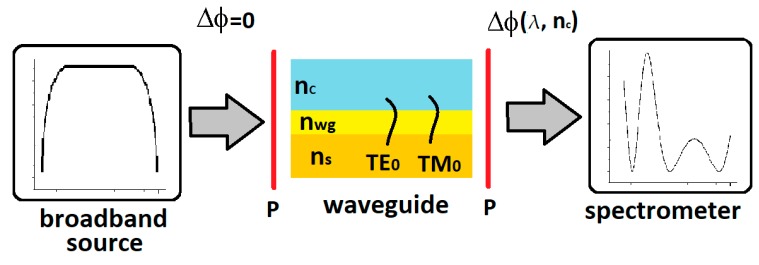
Schematic diagram of a broadband difference interferometer. The system includes a broadband light source, polarizer (P), waveguide (*n_s_*: substrate, *n_wg_*: waveguide layer, *n_c_*: cover), and a spectrometer for recording the output signal.

Figure 1 shows a diagram of a broadband difference interferometer.

Light from a broadband source excites the fundamental optical TE_0_ and TM_0_ modes in a planar waveguide after passing through a polarizer. A phase difference Δ*φ* accumulates between them during propagation. The spectrometer can be used to record an interference signal in the wavelength domain after passing through the output polarizer [29]. If light with the same optical power distribution *I*_0_(λ) is introduced to each of the orthogonal modes TE_0_ and TM_0_, the spectral signal *I*(λ) is given by:

(1) $$I\left(\lambda \right)=\frac{1}{2}I_{0}\left(\lambda \right)\left\{1+\cos\left[\Delta \phi \left(\lambda \right)\right]\right\},$$

where λ is the electromagnetic wavelength. 

The normalized light intensity distribution, at the output *I_n_*, can be defined as follows:

(2) $$I_{n}\left(\lambda \right)=\frac{1}{2}\left\{1+\cos\left[\Delta \phi \left(\lambda \right)\right]\right\},$$

where Δ*φ* is the phase difference between the modes at the waveguide output [29].

**Figure 2:**
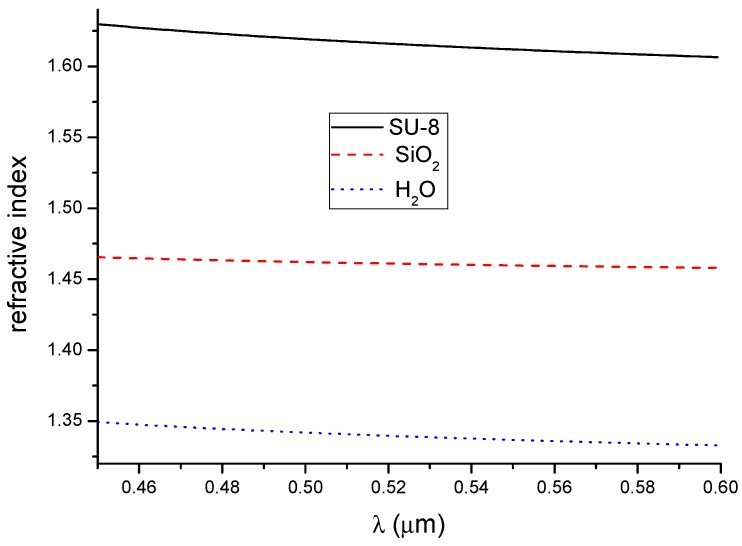
Dispersion in the waveguide structure.

A three-layer system with a SiO_2_ substrate, the SU-8 polymer waveguide layer, and a water (H_2_O) cover layer was used to theoretically analyze the polymer broadband difference interferometer. Optical dispersion in the waveguide layer (SU-8) and substrate (SiO_2_) were taken from ellipsometric measurements presented in the literature [30]. The refractive index dispersion of water was also taken from the literature [40]. The dispersion characteristics are shown in Figure 2. For these calculations, the thickness of the waveguide layer *d* was assumed as a parameter. The thicknesses of the cover and substrate were assumed to be semi-infinite.

Due to available radiation sources and relatively popular waveguide spectrometers, the analysis was carried out for wavelengths ranging from 450 nm to 600 nm. This choice of spectral range may facilitate simpler experimental verification of the proposed system.

## 3. Results and Discussions

In planar asymmetrical three-layer structures where *n_c_* ≠ *n_s_*, propagation of the m-order mode is possible only if the waveguide thickness *d* > *d_c_* [41]. These parameters for TE and TM polarization are described by the following equations [41]:

(3) $$d_{c\;TEm}\left(\lambda \right)=\frac{m\pi +\arctan\left[{\left(\frac{{n_{s}}^{2}-{n_{c}}^{2}}{{n_{wg}}^{2}-{n_{s}}^{2}}\right)}^{1/2}\right]}{\frac{2\pi }{\lambda }{\left({n_{wg}}^{2}-{n_{s}}^{2}\right)}^{1/2}}\;\mathrm{for}\;\mathrm{TE},$$

(4) $$d_{c\;TMm}\left(\lambda \right)=\frac{m\pi +\arctan\left[{\left(\frac{n_{wg}}{n_{c}}\right)}^{2}{\left(\frac{{n_{s}}^{2}-{n_{c}}^{2}}{{n_{wg}}^{2}-{n_{s}}^{2}}\right)}^{1/2}\right]}{\frac{2\pi }{\lambda }{\left({n_{wg}}^{2}-{n_{s}}^{2}\right)}^{1/2}}\;\mathrm{for}\;\mathrm{TM},$$

where *n_c_* is the refractive index of the cover, *n_s_* is the refractive index of the substrate, *n_wg_* is the refractive index of the waveguide layers, and *m* = 0, 1,… is the order of the mode propagating in the waveguide structure [41].

**Figure 3:**
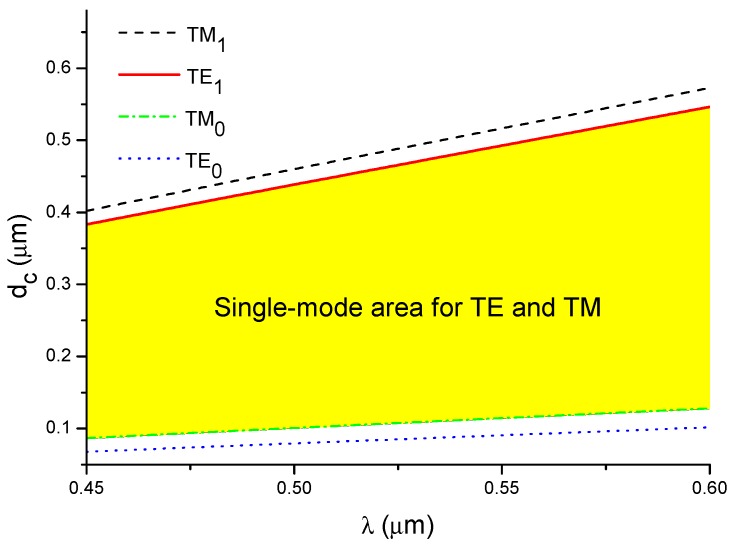
Dependence of the cut-off thickness on wavelength.

The dependence of the cut-off thickness for various modes (fundamental and the first order modes for both polarizations) on the wavelength is presented in Figure 3. This figure also shows a surface whose points determine the layer thickness and the wavelength for which TE_m_ and TM_m_ are the only allowed modes. The main analysis was performed for a waveguide with thicknesses *d*_3_ = 350 nm (Figures 7, 9 and 10) which is a single-mode waveguide in the considered wavelength range. To show the effect of thickness on the characteristics of the interferometer, the characteristics of the waveguides with thickness *d_i_* = *d*_3_ ± 50 nm and *d_j_* = *d*_3_ ± 100 nm are also presented. Five structures with waveguide thicknesses of *d*_1_ = 250 nm, *d*_2_ = 300 nm, *d*_3_ = 350 nm, *d*_4_ = 400 nm, and *d*_5_ = 450 nm were selected for further analysis. Structures with a thickness *d*_4_ = 400 nm and *d*_5_ = 450 nm are not multimode structures over the entire wavelength range, but their characteristics (of the fundamental modes) were included.

**Figure 4:**
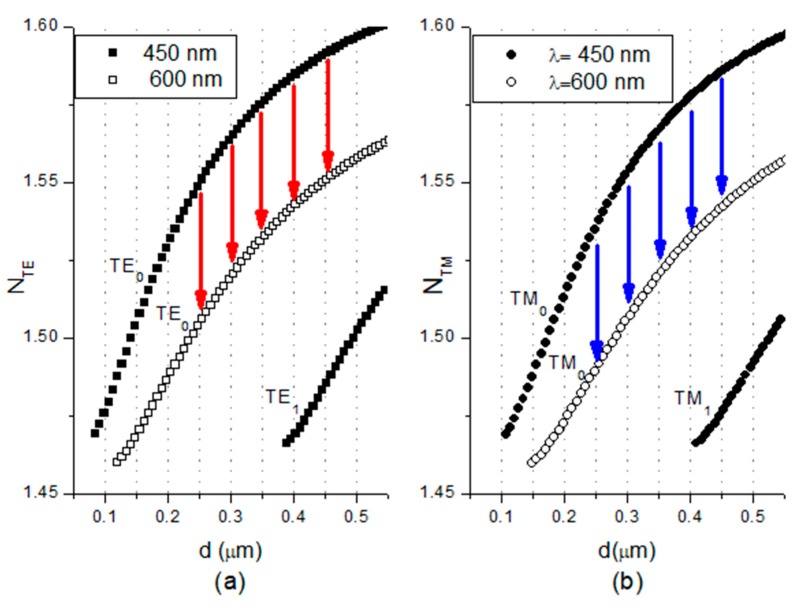
Effective refractive indices (**a**) TE polarization and (**b**) TM polarization as a function of the waveguide layer thickness *d* at 450 and 600 nm.

Figure 4 shows the effective refractive indices for the propagating modes at the minimum (450 nm) and maximum (600 nm) wavelengths in the considered interval as a function of the waveguide layer thickness d. 

Figure 4 shows changes in the effective refractive index for both polarizations that are marked with arrows. The phase difference Δ*φ* between the modes is a function of propagation path length *L*, effective refractive indices (N_TM_ and N_TE_), and wavelength *λ*. This is described by the following equation [29]:

(5) $$\Delta \phi \left(\lambda ,n_{c}\right)=2\pi \frac{\left[N_{\mathrm{TE}}\left(\lambda ,n_{c}\right)-N_{\mathrm{TM}}\left(\lambda ,n_{c}\right)\right]}{\lambda }\cdot L,$$

where *n_c_* is the refractive index of the cover.

The mode propagation constant *β* determines the phase change per unit distance along the propagation path. Therefore, this parameter will be used to describe the broadband interference phenomenon in the next section. Methods for directly measuring and determining differences in the propagation constants Δ*β* can be found in the literature [42,43]. The relationship between the propagation constant and the effective refractive index is expressed as follows:

(6) $$\beta_{i}\left(\lambda ,n_{c}\right)=2\pi \frac{N_{i}\left(\lambda ,n_{c}\right)}{\lambda },$$

after substituting in Equation (3) into Equation (6), one can derive the following:

(7) $$\Delta \phi \left(\lambda ,n_{c}\right)=\Delta \beta \left(\lambda ,n_{c}\right)\cdot L,$$

where Δ*β* is the difference in the propagation constants for TE and TM modes:

(8) $$\Delta \beta \left(\lambda ,n_{c}\right)=\beta_{\mathrm{TE}}\left(\lambda ,n_{c}\right)-\beta_{\mathrm{TM}}\left(\lambda ,n_{c}\right).$$

**Figure 5:**
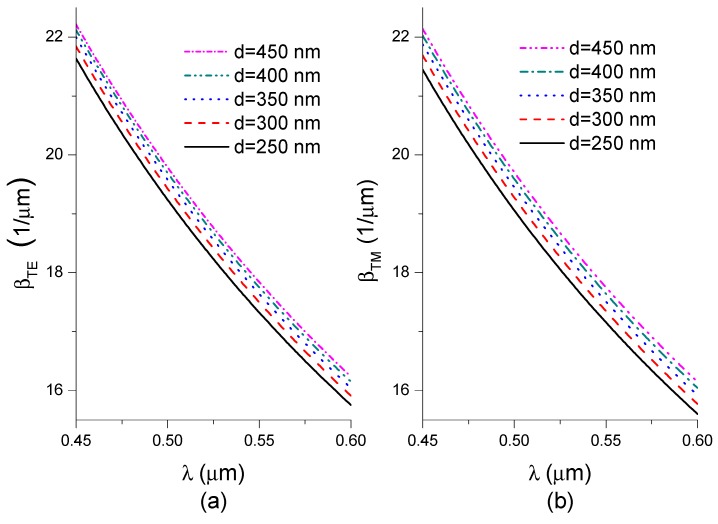
Dependence of the propagation constants on wavelength (**a**) TE polarization and (**b**) TM polarization (SU-8 thickness of 250 nm, 300 nm, 350 nm, 400 nm, and 450 nm).

Figure 5 shows the calculated propagation constants for TE_0_ and TM_0_ modes at the considered waveguide layer thickness as a function of the wavelength.

**Figure 6:**
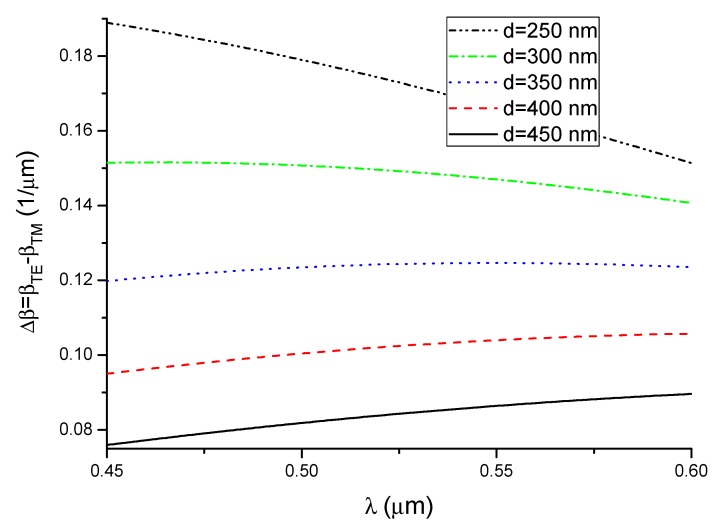
Difference in propagation constants as a function of wavelength.

The propagation constants decrease monotonically in the considered wavelength range for both types of propagating modes. *β* increases as the waveguide thickness increases. It follows from Equation (7) that the shape of the phase difference dependence Δ*φ*(*λ*, *n_c_*), as a function of the wavelength for a given refractive index of the cover n_c_, is determined by the shape of the function describing Δ*β*(*λ*, *n_c_*). The shape of the function Δ*β*(*λ*, *n_c_*) is of key importance for the operation of the interferometric system analyzed here. Figure 6 shows the functions determined for the considered waveguide thickness, where the refractive index of water is denoted *n_c_*_1_.

For the 250 nm thick waveguide, the difference in propagation constants decreases as the wavelength increases. The function Δ*β*(*λ*, *n_c_*_1_) increases until it reaches a maximum value and then starts decreasing in the 300 and 350 nm thick waveguides. For other thickness values (400 nm and 450 nm), the difference in propagation constants increases as the wavelength increases.

**Figure 7:**
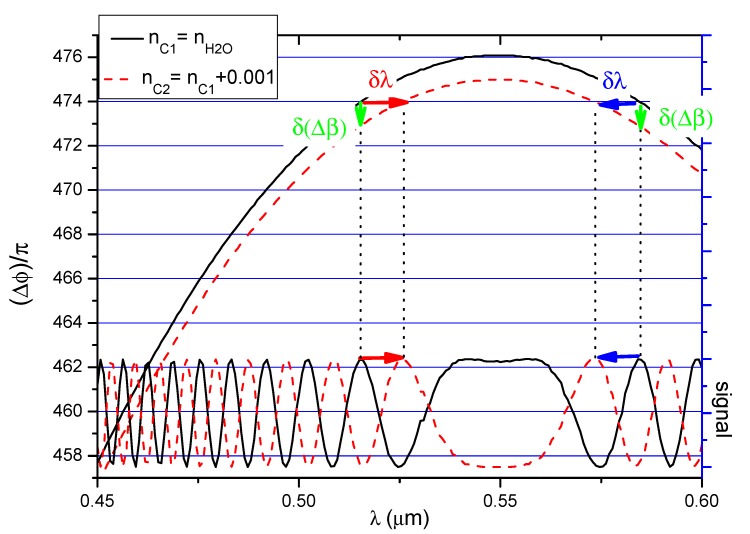
Left vertical axis: phase difference Δ*ϕ* between the modes divided by π for *n_H2O_*(λ) (solid line) and n_c2_(λ) (broken line). Right vertical axis: light intensity distribution for both covers after light propagates 12 mm.

To illustrate the impact of changes in the cover refractive index on the output signal, the 350 nm thick waveguide was selected because the function Δ*β*(*λ*, *n_c_*_1_) is increases and the decreases over the considered wavelength range. Differences in propagation constants for the three-layer system with refractive indices n_SiO2_/n_SU8_/n_H2O_ and n_SiO2_/n_SU8_/n_c2_ were determined when the cover refractive index was increased by 0.001 (i.e., *n_c_*_2_(*λ*) = *n_H2O_*(*λ*) + 0.001). Phase differences between modes were determined using Equation (7). The phase differences for *n_c_*_1_(*λ*) (continuous line) and *n_c_*_2_(*λ*) (broken line) after propagating a distance of 12 mm are shown in the upper part of Figure 7. If the value of the waveguide covers refractive index increases, the phase differences decreases for all wavelengths in the range under consideration. The function Δ*φ*(*λ*, *n_c_*_2_) (broken line) has nearly the same shape as the function Δ*φ*(*λ*, *n_c_*_1_) and is translated vertically along the y axis.

The lower part of Figure 7 shows the output interference signal for the two waveguide covers analyzed here (solid line: water; broken line: *n_c_*_2_).

Let us consider the wavelength range in which the function Δ*φ*(*λ*, *n_c_*_1_) (solid line in the upper part of Figure 7) increases. The maximum value of the output signal occurs when Δ*φ*(*λ*, *n_c_*) is equal to an integer multiple of 2π.

If the cover refractive index increases, the value of the function Δ*φ* (*λ*, *n_c_*) will change (broken line in the upper part of Figure 7), and thus the output signal of the system will also change (broken line in the lower part of Figure 5). To visualize these changes, one maximum was chosen in the considered figure. This maximum was connected to the function Δ*φ*(*λ*, *n_c_*_1_) using a dotted line. For changes in the cover refractive index, the selected maximum shifts towards longer wavelengths.

If we consider the wavelength range in which the function Δ*φ*(*λ*, *n_c_*) decreases, then the extrema of the output signal will shift towards shorter wavelengths as the refractive index increases.

If the function Δ*φ*(*λ*, *n_c_*_1_) is constant over a certain wavelength range (around maximum), then the function Δ*φ*(*λ*, *n_c_*_2_) will also be constant in this interval (albeit with a different value compared to the case with *n_c_*) when the refractive index changes. The interference signal in this interval will change by the same value for each wavelength in this range.

**Figure 8:**
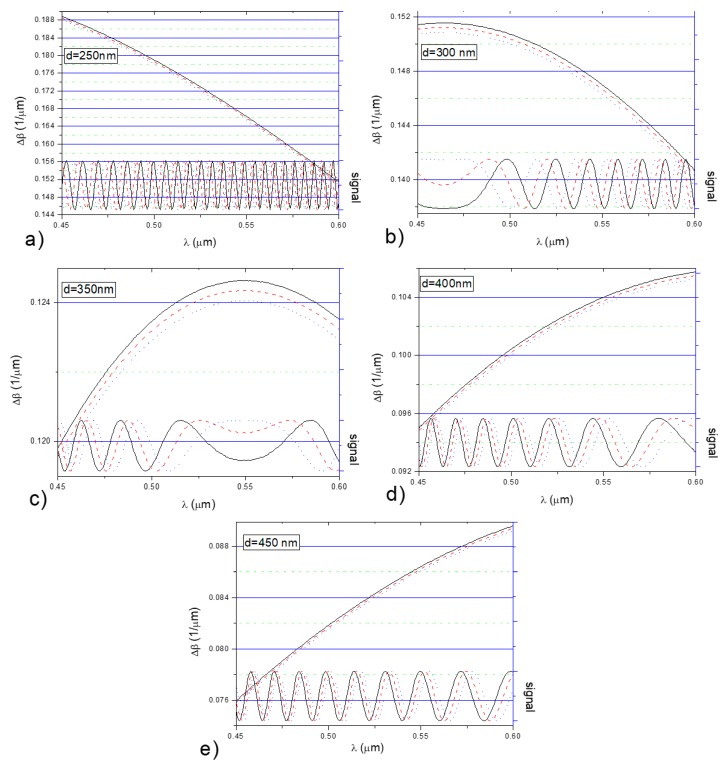
Impact of the cover refractive index change on the functions Δ*β* (left axis) and the output signal (right axis) of the system (for the propagation path length of 4mm), and waveguide thickness: (**a**) 250 nm, (**b**) 300 nm, (**c**) 350 nm, (**d**) 400 nm, (**e**) 450 nm. (Solid black line for *n_c_*_1_(*λ*) = *n_H2O_*, red dash line for *n_c_*_2_(*λ*) = *n_H2O_*(*λ*) + 0.001, blue dot line for *n_c_*_3_(*λ*) = *n_H2O_*(*λ*) + 0.002.)

Figure 8 shows the impact of changes in the cover refractive index on Δ*β*(*λ*, *n_c_*) and the output signal for the previously selected waveguide thickness values (250 nm, 300 nm, 350 nm, 400 nm, and 450 nm). The differences in the propagation constants for the three-layer system were determined with given levels of dispersion in the cover (water), i.e., when *n_c_*_1_(*λ*) = *n_H2O_*(*λ*), *n_c_*_2_(*λ*) = *n_H2O_*(*λ*) + 0.001 (broken line), and *n_c_*_3_(*λ*) = *n_H2O_*(*λ*) + 0.002 (dotted line). 

The distance between extrema of the output signal (oscillation period) in the wavelength domain depends on the slope (derivative) of Δ*β*. If the slope of Δ*β* decreases, the oscillation period increases. The shift of the signal extrema due to an increase in the cover refractive index is determined by the monotonicity of Δ*β*.

**Figure 9:**
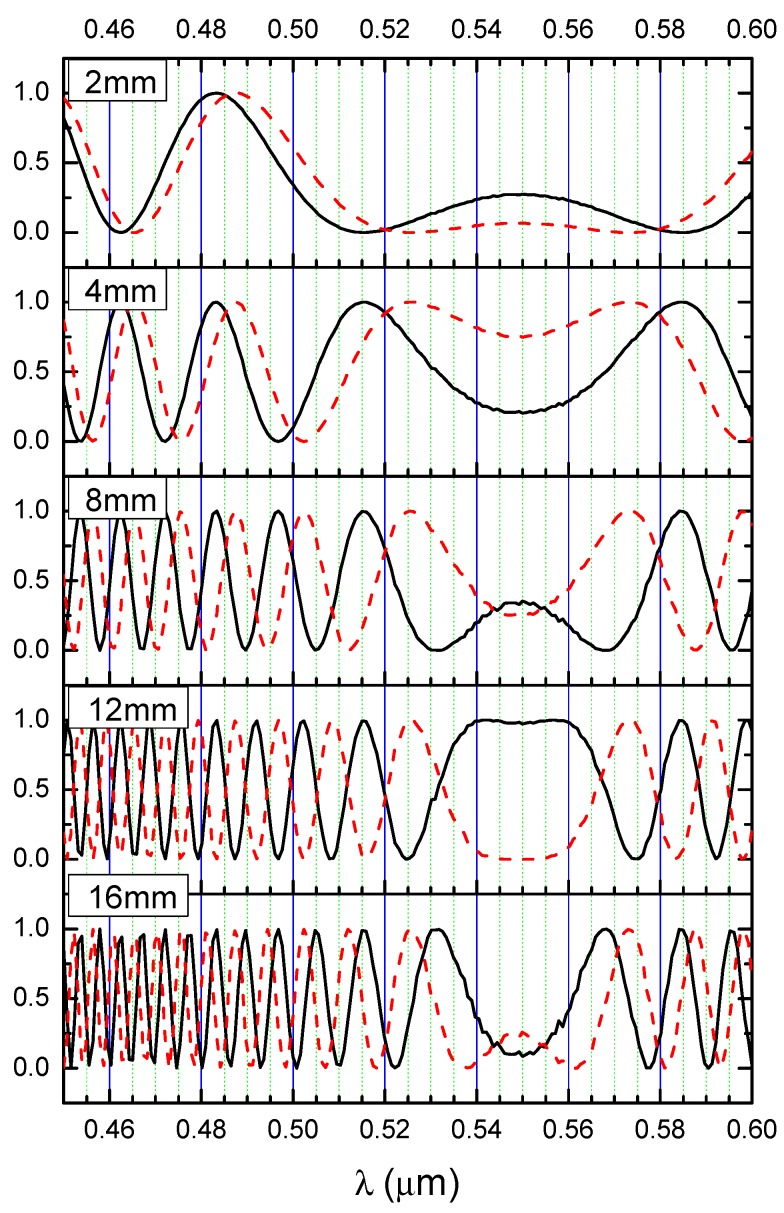
Light intensity distribution for different length of the propagation path. Solid line for the cover n_H2O_, broken line for the cover *n_c_*_2_. (Solid black line for *n_c_*_1_(*λ*) = *n_H2O_*, red dash line for *n_c_*_2_(*λ*) = *n_H2O_*(*λ*) + 0.001.)

According to Equation (1) and (7), the oscillating output signal depends on the length of propagation path *L* in the structure. Figure 9 shows the calculated signal as a function of the wavelength when *d* = 350 nm for the propagation path lengths of *L*_1_ = 2 mm, *L*_2_ = 4 mm, *L*_3_ = 8 mm, *L*_4_ = 12 mm, and *L*_5_ = 16 mm.

The phase difference increases as the propagation path increases linearly for each wavelength according to Equation (5), which increases the number of recorded extrema. It is worth noting that, just like the case of a broadband Mach–Zhender interferometer [5], the shift in the extrema does not depend on the length of the propagation path.

**Figure 10:**
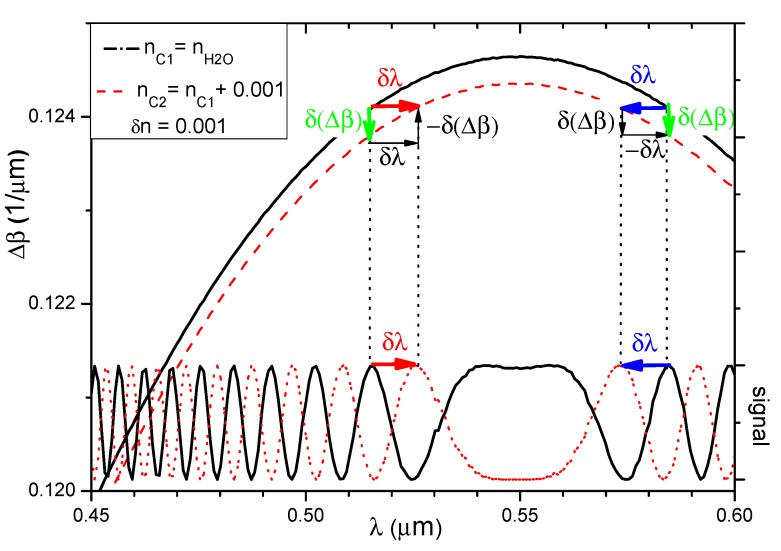
Left vertical axis: propagation constants difference Δβ between the modes for *n_H2O_* (solid line) and *n_c_*_2_ (dash line). Right vertical axis: light intensity distribution for both covers after light propagates 12 mm.

Figure 10 shows the functions Δ*β*(*λ*, *n_c_*) and interference signal. Assuming the equality of partial derivatives of the Δ*β*(*λ*, *n_c_*) function with respect to the wavelength for the cover refractive index *n_c_*_1_ and *n_c_*_2_ = *n_c_*_1_ + *δn* (*δn*, non-significant change in the refractive index) for the same λ values, one can write:

(9) $$\frac{\partial \left(\Delta \beta \left(\lambda ,n_{c}+\delta n\right)\right)}{\partial \lambda }\approx \frac{\partial \left(\Delta \beta \left(\lambda ,n_{c}\right)\right)}{\partial \lambda }.$$

As shown in Figure 10, the quotient of the change *δ*(Δ*β*) of the difference of propagation constants divided by the change of the location of extremum *δλ* can be associated with the partial derivative of the function Δ*β*(*λ*, *n_c_*) with respect to the wavelength by the relationship:

(10) $$\frac{\delta (\Delta \beta )}{\delta \lambda }\approx -\frac{\partial (\Delta \beta )}{\partial \lambda },$$

**Figure 11:**
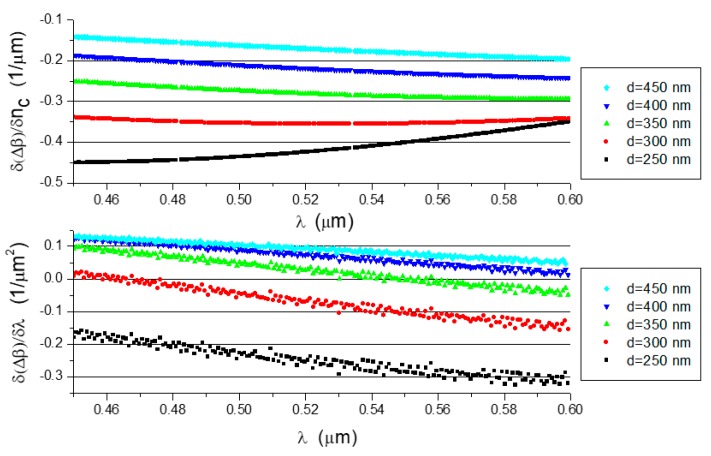
$\frac{\partial (\Delta \beta )}{\partial n_{c}}$ (**top**) and $\frac{\partial (\Delta \beta )}{\partial \lambda }$ (**bottom**) determined for the analyzed waveguide structures.

*δ*(Δ*β*) can be expressed by the partial derivative of the function Δ*β*(*λ*, *n_c_*) with respect to the cover refractive index and the change of the cover refractive index *δn_c_*:

(11) $$\delta (\Delta \beta )\approx \frac{\partial (\Delta \beta )}{\partial n_{c}}\times \delta n_{c}.$$

taking into account (9), after substituting in (10) the expression (11) and transforming, one obtains:

(12) $$\delta \lambda \approx -\frac{\frac{\partial (\Delta \beta )}{\partial n_{c}}}{\frac{\partial (\Delta \beta )}{\partial \lambda }}\times \delta n_{c}.$$

the numerically-determined derivatives $\frac{\partial (\Delta \beta )}{\partial n_{c}}$ and $\frac{\partial (\Delta \beta )}{\partial \lambda }$ for the analyzed waveguide structures are shown in Figure 11. The derivatives $\frac{\partial (\Delta \beta )}{\partial n_{c}}$ are slowly changing functions with negative values in the analyzed domain.

The derivatives $\frac{\partial (\Delta \beta )}{\partial \lambda }$ are approximately linear and they have small values for some waveguide thicknesses, including a zero value.

**Figure 12:**
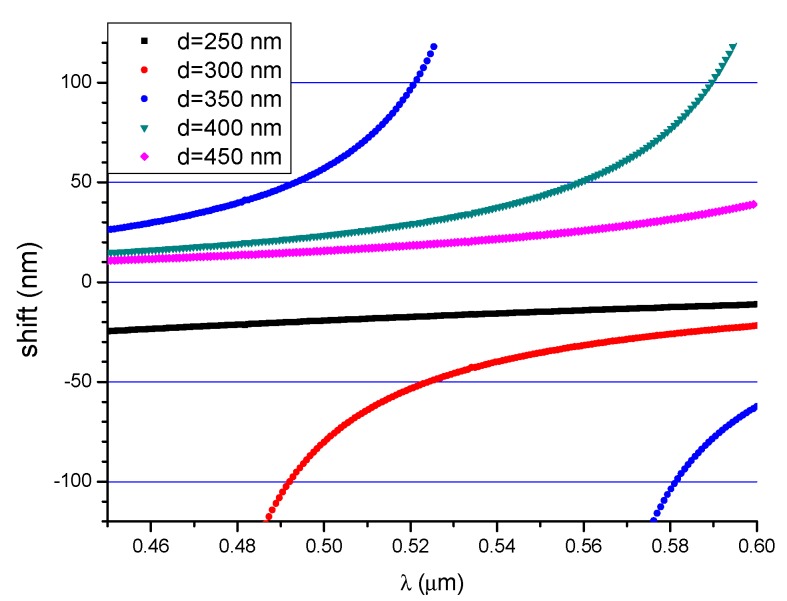
The shifts in the extrema as a function of wavelength for different waveguide thicknesses.

The shifts in the extrema *δλ* for structures with the considered waveguide thickness were determined using Equation (12), and the results are shown in Figure 12. The change in the refractive index was set to *δn_c_* = 0.01 [29] in order to compare the SiO_2_/Si_3_N_4_/H_2_O structures.

The negative shift in the extrema (blue shift) is obtained over the entire wavelength range of the 250 nm thick waveguides. Both types of shifts (red and blue shift) are observed (some points are not visible due to their high values) in structures with thickness values of 300 and 350 nm. The shift of extrema δλ diverges to infinity as the value of the function $\frac{\partial (\Delta \beta )}{\partial \lambda }$ tends to zero. A positive shift in the output signal extrema (red shift) is obtained over the whole range for waveguides with thickness values of 400 and 450 nm. 

**Figure 13:**
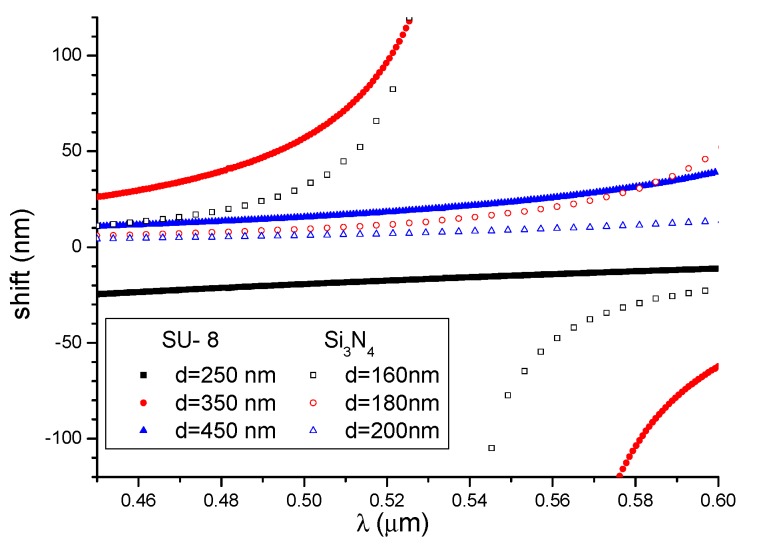
Shifts in the maxima for the SU-8 and Si_3_N_4_ waveguides.

Figure 13 shows the shifts in the maxima for the SU-8 and Si_3_N_4_ waveguides as the cover refractive index was increased by 0.01 (data for Si_3_N_4_ waveguides was taken from the literature [29]). Three thickness values of the structure were selected for each material. As can be seen in the figure, a higher maximum shift can be obtained for polymer waveguides as compared with Si_3_N_4_ based waveguides. According to Equation (11), a small value of $\frac{\partial (\Delta \beta )}{\partial \lambda }$ in the denominator of Equation (11) causes a relatively large shift of the spectrum maxima at the output, despite the smaller value of $\frac{\partial (\Delta \beta )}{\partial n_{c}}$ in polymer waveguides.

The experimental implementation of a broadband differential interferometer for a waveguide with low contrast of refractive indexes has been presented in [44]. The visible spectrum light (450–750 nm) was introduced into the waveguide from the xenon lamp. The interference signal was obtained after passing the *L* = 20 mm propagation path through the TE_0_ and TM_0_ modes. It has been shown that the change in the refractive index of the cover causes the registered maxima of the interference signal to be shifted only for one waveguide. However, the impact of geometric parameters of waveguides on the recorded interference signal has not been considered in this work.

## 4. Conclusions

Polymer planar waveguides, due to the relatively low cost of manufacture, but also the possibility of obtaining a relatively low attenuation of the optical modes are used in the technology of sensors. The polymer SU-8 is a material that is frequently used in the technology of MEMS and also for the construction of waveguide interferometer systems. The model for a broadband difference interferometer based on the polymer SU-8 shows that the waveguide layer thickness has a significant influence on the output signal from the system. For the selected thickness (e.g., 250 nm, 400 nm, and 450 nm), the sinusoidal output signal shifts monotonically as the cover refractive index changes. An algorithm for determining a change in the phase difference [16] can be used with systems that include this type of waveguide. For other waveguide thicknesses (e.g., 300 nm and 350 nm), there is the possibility of detection described in [17] when a change in the cover refractive index causes the same change in the phase difference at the system output within a selected wavelength range. The shift in the extrema in the output signal is directly proportional to $\frac{\partial (\Delta \beta )}{\partial n_{c}}$ and inversely proportional to $\frac{\partial (\Delta \beta )}{\partial \lambda }$. A relatively large shift of extrema is obtained in waveguides with thicknesses at which the derivative in the denominator reaches low values. The determined shifts in the output signal extrema for polymer waveguides are comparable, and these shifts are larger for some waveguide thicknesses compared to waveguides based on Si_3_N_4_.
